# Compact Second-Harmonic Generation in the C‑Exciton Band of 3R-MoS_2_ for Integrated Quantum Photonics

**DOI:** 10.1021/acsphotonics.5c01266

**Published:** 2025-12-15

**Authors:** Alessandro Bile, Domenico de Ceglia, Daniele Ceneda, Maria Cristina Larciprete, Marco Centini

**Affiliations:** † Department of Basic and Applied Science for Engineering (SBAI), 9311 SAPIENZA, Università di Roma, Via Scarpa, 16, Roma 00161, Italy; ‡ Department of Information Engineering, 9297 University of Brescia, Via Branze 38, Brescia 25123, Italy

**Keywords:** Second harmonic generation, Nonlinear 2D materials, Hybrid frequency converters, Integrated quantum photonics

## Abstract

We present a novel scheme for highly efficient second-harmonic generation in the visible spectrum using minimal volumes of 3R-phase molybdenum disulfide (MoS_2_) integrated on a silicon nitride (Si_3_N_4_) ridge waveguide. The device is designed to operate near the C-exciton resonance of MoS_2_, where the material exhibits its strongest second-order nonlinear response. A periodic array of nanometric MoS_2_ stripes is patterned on the waveguide surface to form a one-dimensional photonic crystal, enabling strong field localization at the pump wavelength (λ_FF_ ≈ 890 nm) near the photonic band edge. This leads to resonant second-harmonic emission around 445 nm, close to the highly absorbing C-exciton. To mitigate the absorption while maintaining phase matching, we implement a counter-propagating pump configuration that ensures zero in-plane momentum mismatch and enables vertical out-coupling of the second harmonic signal via a leaky-mode resonance. The combination of strong nonlinear susceptibility, mode engineering, and resonant coupling allows for efficient frequency conversion within an interaction region only tens of nanometers thick, with an overall device footprint on the order of 1 μm^2^. Full-wave 3D simulations predict conversion efficiencies up to 0.004% at peak pump intensities of 300 MW/cm^2^. Moreover, the same architecture is compatible with spontaneous parametric down-conversion (SPDC), enabling integrated photon-pair generation with intrinsic modal and directional filtering. These features make the platform well-suited for compact, scalable nonlinear, and quantum photonic applications in the visible and near-infrared range.

## Graphical abstract

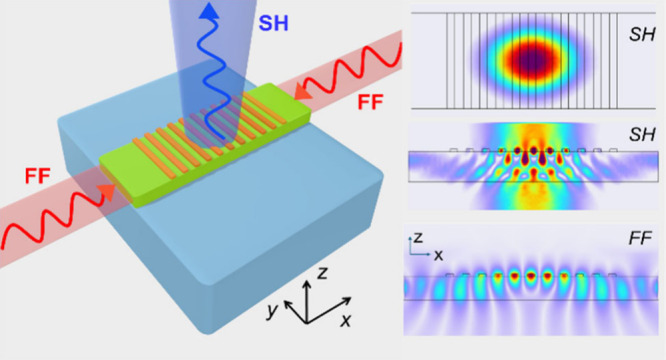

## Introduction

Second-harmonic generation (SHG) in two-dimensional (2D) materials has attracted intense interest due to the interplay of atomic thickness, reduced symmetry, and strong light–matter interaction.^1,2^ Among transition metal dichalcogenides (TMDs), molybdenum disulfide (MoS_2_) in the 2H phase has been extensively studied. In its monolayer form, 2H-MoS_2_ lacks inversion symmetry and thus allows electric-dipole SHG, with the nonlinear response significantly enhanced near excitonic resonances.^3–5^ These enhancements arise from the strong light–matter interactions associated with the A, B, and C excitons, located respectively at approximately 1.88 eV (∼660 nm), 2.03 eV (∼610 nm), and 2.8–2.9 eV (∼440 nm).^6,7^ However, the SHG signal in MoS_2_ drops dramatically when moving beyond the monolayer, due to the restoration of centrosymmetry in the 2H-stacked multilayer and bulk crystals. The 2H polytype belongs to the *D*_*6h*_ point group, where the alternating 180° rotation between adjacent layers results in global inversion symmetry and suppresses second-order processes.^8^ To improve SHG efficiency in monolayer and few-layer 2H-MoS_2_, several strategies have been proposed. These include coupling with dielectric metasurfaces,^9^ the use of high-Q resonances such as bound states in the continuum (BICs),^10^ and mode phase matching via patterned silicon waveguides.^11^ Despite these efforts, the atomic thickness inherently limits the interaction volume, and the optical field confinement remains suboptimal, hindering the scalability and efficiency of nonlinear processes.

To address these limitations, researchers have recently revisited the lesser-known 3R polytype of MoS_2_,^12^ whose second-order nonlinear properties were originally investigated decades ago.^13^ Unlike 2H-MoS_2_, the 3R phase exhibits a rhombohedral stacking configuration where adjacent layers are shifted but not rotated, thus remaining noncentrosymmetric regardless of the number of layers, enabling bulk-like SHG responses while preserving the strong excitonic enhancement. Recent work has demonstrated the extraordinary nonlinear optical properties of 3R-MoS_2_ nanostructures, including ultrahigh χ^(2)^ values and resonant enhancement near excitonic features.^14^ In this context, several strategies have been extended to 3R-MoS_2_ to further enhance SHG. For instance, Trovatello et al. demonstrated quasi-phase-matched up- and down-conversion in periodically poled stacked structures at telecom wavelengths.^15^ Other approaches include the integration of 3R-MoS_2_ with all-dielectric metasurfaces to exploit metasurfaces that support resonant enhancement,^16^ and high-Q photonic structures exploiting quasi-BIC modes.^17,18^

Several of the aforementioned strategies have also been investigated for their potential in enabling spontaneous parametric down-conversion^19^ (SPDC). SPDC is the quantum-optical counterpart of SHG, whereby a single high-energy photon spontaneously splits into a pair of lower-energy photons, commonly referred to as signal and idler. This process is a cornerstone of quantum photonics, as it provides a reliable source of entangled or correlated photon pairs for applications in quantum communication, quantum computation, and quantum metrology.

Despite these promising results, a fundamental challenge remains: the same excitons that enhance the nonlinear response also introduce significant linear absorption when the fundamental or second-harmonic (SH) wavelengths are resonant with them. This dual role of excitons imposes a severe trade-off. For example, in telecom-oriented applications, where the pump wavelength is near 1550 nm, the generated SH lies close to the visible range, around 775 nm, and can be spectrally close to both the A and B excitons of MoS_2_.^6,7^ In this case, moderate absorption can affect SH extraction efficiency. The situation becomes more critical moving toward the visible regime, where the nonlinear susceptibility χ^(2)^ is maximized. According to Zograf et al.,^14^ the strongest nonlinear response, with an outstanding χ^(2)^ value of about 800 pm/V, occurs for a pump tuned around 890 nm, yielding an SH near 445 nm, which is nearly resonant with the C-exciton, the strongest and most absorbing excitonic transition. In this regime, the absorption is so pronounced that phase-matching strategies requiring extended interaction volumes, such as quasi-phase matching, become ineffective, as even tens of nanometers of 3R-MoS_2_ introduce substantial attenuation. This underscores the need for alternative schemes that minimize the interaction volume while preserving high field intensities.

In this work, we propose a novel scheme for efficient second-harmonic generation (SHG) in the visible spectrum, which leverages minimal volumes of 3R-MoS_2_ to mitigate absorption losses. The proposed device is based on silicon nitride (Si_3_N_4_) ridge waveguides fabricated on a SiO_2_ substrate. The top surface of the waveguide is periodically patterned with MoS_2_ stripes, each with a thickness and width of a few tens of nanometers. These stripes are carefully dimensioned and spaced to align the pump field with the band-edge (BE) frequency of the resulting one-dimensional photonic crystal, where strong field localization enhances the nonlinear interaction.^20^ Furthermore, the spatial distribution of the pump field is coherently controlled using counter-propagating waveguided modes. This counter-propagating scheme was originally introduced decades ago in planar semiconductor waveguides for second-order nonlinear processes, as it inherently satisfies in-plane phase matching conditions by ensuring that the total in-plane linear momentum is zero.^21^ As a result, the generated second-harmonic signal is emitted out of plane, orthogonally to the waveguide surface, with high directionality.

While vertical SHG in 2D materials integrated with photonic crystal waveguides has been recently investigated,^22^ those approaches typically rely on a single monolayer or thin flake coupled to the waveguide. In contrast, our strategy leverages multiple patterned MoS_2_ elements, and combines four key mechanisms, i.e. BE field enhancement of the pump, counter-propagating waveguided excitation, the intrinsically high second-order nonlinearity of MoS_2_, and resonant coupling of the SH signal to a leaky-mode emitting light in the vertical direction. This configuration supports highly efficient nonlinear interactions within ultrathin active volumes, maintaining a total device footprint of approximately 1 μm^2^. By localizing the nonlinear response exclusively within the patterned MoS_2_ elements, the design suppresses parasitic effects in the Si_3_N_4_ waveguide and allows for precise spatial control of the nonlinear region, while the surrounding platform remains entirely linear. When coherently excited by counter-propagating guided pump modes tuned at 890 nm, the device acts as an efficient, micron-sized SHG source emitting vertically with a well-defined, Gaussian-like beam profile. Our full-wave 3D numerical simulations predict second-harmonic conversion efficiencies as high as 0.004% for pump intensities around 300 MW/cm^2^.

Importantly, the proposed architecture is also highly suitable for the implementation of spontaneous parametric down-conversion, as the generated photon pairs are intrinsically coupled into the guided modes of the ridge waveguide. Analogous configurations based on planar GaAs waveguides have been previously employed for photon-pair generation.^23,24^ In our case, the use of a laterally confined ridge geometry, combined with a spatially localized nonlinear interaction region, enables intrinsic modal and directional filtering of the emitted photon pairs. This not only ensures high overlap with the pump and guided modes but also facilitates direct integration with downstream on-chip quantum photonic components. Furthermore, as previously mentioned, the strong nonlinear response of 3R-MoS_2_ allows for efficient pair generation within an interaction volume of subwavelength dimensions, while the surrounding Si_3_N_4_ platform remains strictly linear, eliminating parasitic nonlinearities. These characteristics position our device as a highly compact, integrable, and scalable source of photon pairs, particularly advantageous in the visible-to-near-infrared spectral range where silicon-based single-photon detectors exhibit optimal quantum efficiency.

## Methods

### Optimization of Pump Field’s Localization

We consider a ridge waveguide geometry based on a silicon nitride (Si_3_N_4_) core on top of a silicon dioxide (SiO_2_) substrate. The waveguide has a width of approximately 1 μm and a thickness of 310 nm. These dimensions are commonly adopted in visible and near-infrared integrated photonics platforms due to their fabrication compatibility and low propagation losses. At the pump, i.e., at the fundamental-frequency (FF) wavelength λ_FF_ = 890 nm, numerical simulations indicate that the unpatterned structure supports a TE-like mode, with the electric field mostly *y*-polarized and localized inside the Si_3_N_4_ core region.^25^ We focus on this wavelength in order to maximize the value of the second-order nonlinear susceptibility.^14^ Additionally, using *y*-polarized pumps and assuming *y*-polarized SH emission ensures that the nonlinear interaction with 3R-MoS_2_ is governed by the χ_*yyy*_^(2)^ tensor element, which has the largest value. Figure 1 depicts a sketch of the investigated system.

**Figure 1:**
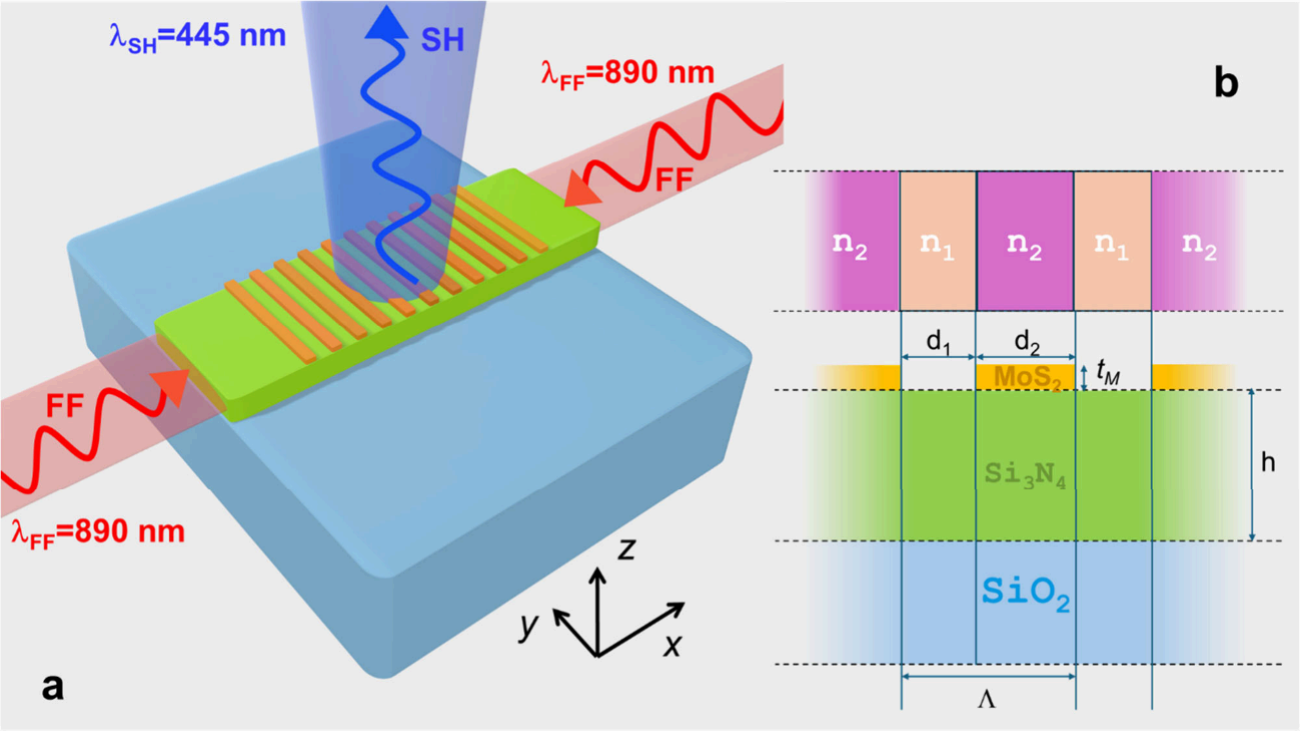
(a) Conceptual illustration of the proposed scheme for vertical SHG in a Si_3_N_4_ waveguide patterned with periodic 3R-MoS_2_ rods, excited by counter-propagating guided pump beams. The periodic structure enables field localization at the photonic band edge and enhances second-harmonic extraction efficiency. (b) Cross-sectional view of the structure and key geometric parameters. In the 1D analytical “toy” model, the system is treated as a photonic crystal with alternating effective indices n_1_ and n_2_ for MoS_2_-free and MoS_2_-covered regions, respectively. The unit cell has period Λ*=d*_*1*_ *+d*_*2*_, MoS_2_ thickness *t*_*M*_, and Si_3_N_4_ waveguide height *h*.

As a first-order model, we treat the structure as a one-dimensional (1D) photonic crystal composed of alternating segments of Si_3_N_4_ waveguide with and without MoS_2_ coverage. In this simplified “toy model,” we approximate each segment as a homogeneous dielectric slab with an effective refractive index: one corresponding to the bare waveguide, and one to the MoS_2_-covered portion (see Figure 1b). This periodic modulation induces a photonic band structure, which can be analyzed semianalytically via transfer-matrix formalism. The periodicity provides a dual advantage: (i) it enhances the electric fields at the photonic BE, where the density of optical states is high and slow-light effects may emerge, and (ii) it minimizes the overall amount of MoS_2_ material used, thereby limiting absorption losses at the second-harmonic wavelength. This enables efficient nonlinear interaction within a subwavelength active region while preserving the low-loss linear propagation properties of the Si_3_N_4_ platform.

Before analyzing the modal dispersion of the 1D photonic crystal structure, we first investigate how the complex effective index of the fundamental TE mode at λ = 890 nm depends on the MoS_2_ layer thickness *t*_*M*_.^26,27^ Figure 2a shows the calculated effective index as a function of *t*_*M*_, with the real part plotted on the left axis (blue line) and the imaginary part on the right (red line). Starting from approximately 1.739 for the MoS_2_-free waveguide, the real part of the effective index increases rapidly with MoS_2_ thickness, reaching ∼ 2.9 for *t*_*M*_ = 50 nm and approaching ∼ 3.7 at 100 nm. This strong contrast is favorable for field localization at the photonic BE. However, the imaginary part, initially negligible, also increases with thickness, reaching ∼ 0.06 for *t*_*M*_ ≈50 nm. While an initial increase in MoS_2_ thickness (*t*_*M*_) up to approximately 30 nm enhances optical confinement and nonlinear overlap, further increases result in substantial absorption losses that outweigh the benefits of increased index contrast. Figures 2b and 2c show the simulated fundamental mode profiles for the uncoated and MoS_2_-loaded waveguide regions, respectively. For *t*_*M*_ = 30 nm, the electric field in the loaded segment is already predominantly confined within the MoS_2_ layer, indicating strong interaction with the nonlinear medium. Based on this analysis, we restrict our optimization to a thickness range between 20 and 50 nm, which provides improved field confinement without incurring excessive optical losses.

**Figure 2:**
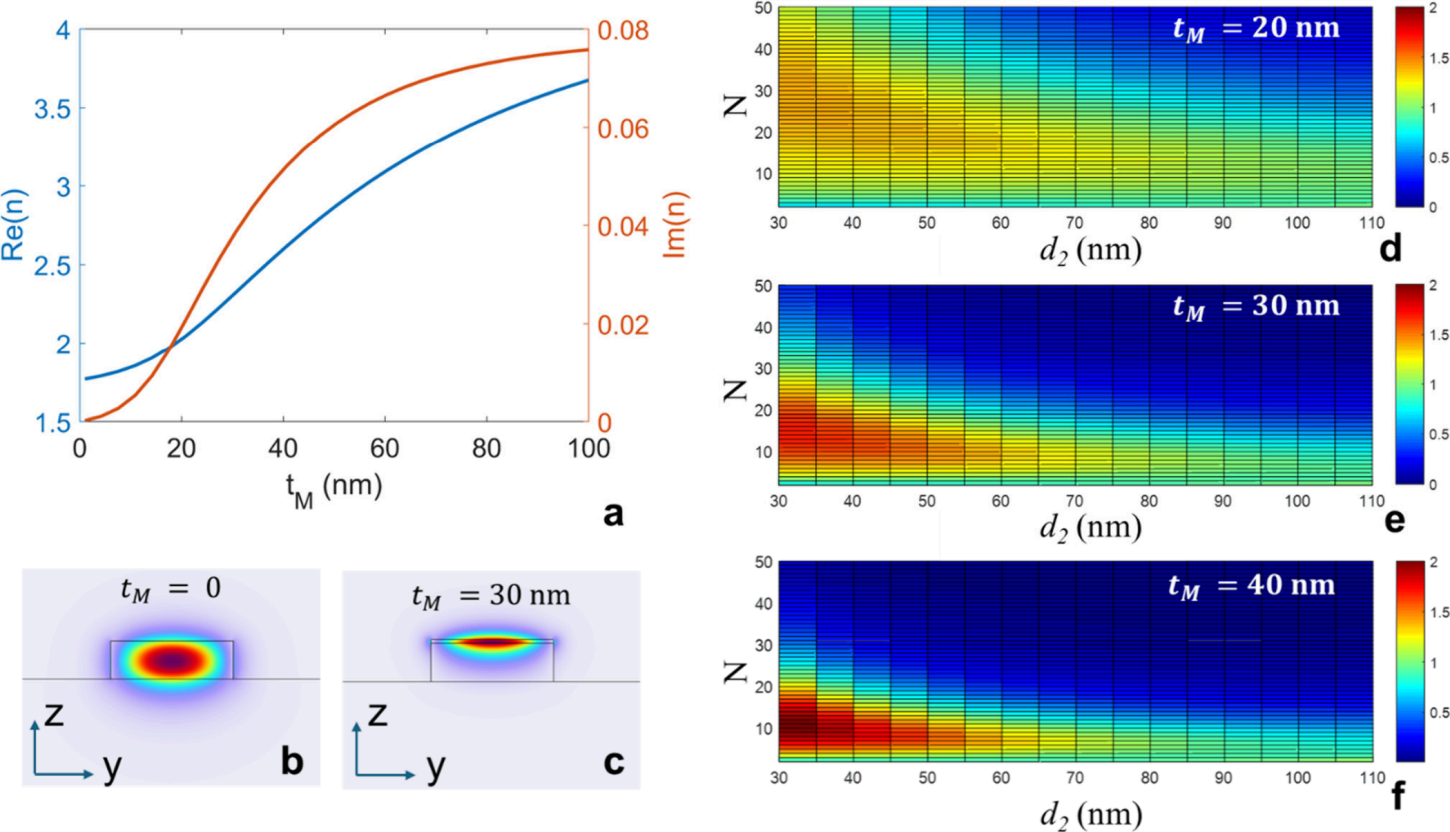
(a) Real (blue, left axis) and imaginary (red, right axis) parts of the effective index of the guided mode as a function of MoS_2_ thickness *t*_*M*_. (b, c) Cross-sectional electric field intensity profiles of the fundamental TE mode for a bare waveguide (*t*_*M*_ = 0) and with a 30 nm MoS_2_ layer. (d–f) Normalized average electric energy density inside the MoS_2_ rods as a function of their length *d*_*2*_ and the number of periods *N*, for *t*_*M*_ = 20 nm (d), 30 nm (e), and 40 nm (f).

Continuing with the toy model, we consider a 1D photonic crystal composed of slabs having different effective propagation constants *β*_*1*_ and *β*_*2*_, referring to the bare Si_3_N_4_ waveguide and the MoS_2_-loaded region, respectively:

1 $$\beta_{1}=\frac{2\pi }{\lambda_{FF}}n_{1};\beta_{2}=\frac{2\pi }{\lambda_{FF}}n_{2}$$

where *n*_*1*_ and *n*_*2*_ are the effective indices evaluated in Figure 2 for the bare waveguide (*t*_*M*_ = 0) and for the MoS_2_-loaded elements, respectively. Using the transfer-matrix approach,^25^ the Bloch condition reads:

2 $$\cos\left(K_{B}\Lambda \right)=\cos\left(\beta_{1}d_{1}\right)\cos\left(\beta_{2}d_{2}\right)-\frac{1}{2}\left(\frac{\beta_{1}}{\beta_{2}}+\frac{\beta_{2}}{\beta_{1}}\right)\sin\left(\beta_{1}d_{1}\right)\sin\left(\beta_{2}d_{2}\right)$$

Here, *K*_*B*_ is the Bloch wave-vector, *d*_*1*_ and *d*_*2*_ denote the lengths of the bare and MoS_2_-covered segments, respectively, and Λ*=d*_*1*_ *+d*_*2*_ is the period of the structure.

In the case of an infinite periodic system, the photonic BE occurs at *K*_*B*_ Λ=π. However, unlike ideal infinite structures, real devices feature a limited number of periods *N* and a discretized set of resonances in the passband. The resonance condition at the BE can be evaluated by the following analytic expression:^28^

3 $$K_{B}\Lambda =\pi \frac{N-1}{N}$$

Equations 2 and 3 allow us to determine the values of *d*_*1*_ and *d*_*2*_ for which the BE resonance occurs at the target wavelength, for a given number of periods *N* and a given value of *t*_*M*_. Once these parameters are identified, a simplified 1D model is employed to study field enhancement in corrugated waveguides and multilayer structures. In this approach, the waveguide is approximated as a multilayer stack, where each layer is characterized by the effective refractive index of the corresponding guided mode.

The structure is illuminated by a monochromatic plane wave incident from one side, with a fixed amplitude *E*_*0*_, which serves as a normalization reference. The internal electric field distribution is calculated using the standard transfer matrix method (TMM).^25^ This model isolates the key physical mechanisms governing field enhancement, such as the role of optical absorption near the band-edge resonance, while neglecting other effects like scattering losses, in-plane propagation, and coupling efficiency variations, which are beyond the scope of this simplified approach. Although it does not provide fully quantitative predictions for realistic devices, it offers valuable intuitive insights and highlights the limiting impact of absorption on field localization near the photonic band edge.

In a lossless finite-size 1D photonic crystal operating near the band-edge (BE) the interference of forward and backward propagating waves due to multiple reflections at interfaces gives rise to a quasi-standing wave, whose envelope typically resembles a Gaussian centered in the structure^29^ and with the electric field intensity enhancement scaling with *N* ^*2*^.^30,31^ This is the optimal condition for a well-defined nonlinear interaction area between FF and SH fields. However, the strong absorption of MoS_2_ limits this enhancement, leading to a trade-off between the amount of MoS_2_ (i.e., the filling ratio) and the number of periods. To better illustrate the behavior of the electric field pattern at the BE resonance, Figure S1 in the Supporting Information shows the squared modulus of the electric field normalized to the squared modulus of the incident field *E*_*0*_, for a representative case with *t*_*M*_ = 20 nm. The figure compares the case including optical absorption (red line) in the high-index material with the ideal lossless case (blue line). In the presence of absorption, the field localization initially increases with the number of unit cells *N*, reaching a maximum before decreasing for larger *N* due to the absorption-induced suppression of the BE resonance. Conversely, in the ideal lossless case, the field intensity continues to increase with *N*.

To quantify this and to have a first indication on the optimal number of unit cells we solve eq 2 under the BE resonance condition of eq 3. For each pair (*d*_*2*_ *,N*), we determine the corresponding *d*_*1*_ that satisfies the BE resonance tuning at λ_FF_ = 890 nm. We then evaluate the field profile by assuming an input field of plane wave monochromatic with amplitude E_0_ and evaluate the following figure of merit:

4 $$W\left(d_{2},N\right)=\frac{1}{N\Lambda }\int_{MoS_{2}}{\left|\frac{E\left(x\right)}{E_{0}}\right|}^{2}dx$$

which is proportional to the average normalized electric energy density stored in the MoS_2_ regions. Maximum values of *W* correspond to maximum overlap of the electric field square modulus in the nonlinear material, and thus to an optimized condition nonlinear interaction.

Figures 2d, 2e, and 2f report the computed values of *W­(d*_*2*_ *,N)* for three different MoS_2_ thicknesses (*t*_*M*_ = 20, 30, and 40 nm), plotted as functions of *N* and *d*_*2*_. We observe that because of the absorption losses, the field enhancement is not monotonically increasing with the number of periods *N*. Indeed we note that the value of *W­(d*_*2*_ *,N)* saturates to a maximum value which is weakly dependent on the MoS_2_ layer thickness *t*_*M*_. Thus, increasing the MoS_2_ layer thickness reduces the optimal number of periods needed to achieve maximum field enhancement, due to stronger index contrast. A representative set of one-dimensional plots showing the dependence of *W­(d*_*2*_ *,N)* on the number of periods *N*, for selected values of *d*_*2*_ = 20, 30, 40, 50 nm, and three different MoS_2_ thicknesses (*t*_*M*_ = 20, 30, 40 nm), is provided in Figure S2 of the Supporting Information. These plots highlight the parameter ranges for which *W* > 1, corresponding to an effective pump field enhancement within the nonlinear material.

While this simplified model does not account for radiation or scattering losses, nor for coupling to higher-order modes of the loaded and bare waveguide segments, and cannot predict the real electric field mode in the ridge waveguide, it offers valuable design insight for the tuning of the pump fields. It suggests an optimal design range of 10–20 periods, MoS_2_ thicknesses between 20 and 40 nm, and segment lengths *d*_*2*_ ranging from 40 to 120 nm. The corresponding values of d_1_ required for BE tuning range approximately from 190 to 70 nm making the optimal lattice constant Λ varying in the range from 180 to 230 nm.

### The Counter-Propagating Pump Scheme

We propose a counter-propagating pump configuration to address the intrinsic phase mismatch between the fundamental frequency (FF) and second harmonic (SH) fields, while simultaneously enabling highly directional SH emission from a compact interaction region. This strategy, originally introduced in the context of nonlinear waveguides,^21^ inherently satisfies the phase-matching condition along the horizontal (*x*-) direction when SH light is emitted vertically. In particular, when two coherent pump beams are launched in opposite directions along the *x*-axis, their in-plane momenta cancel each other, i.e., (*k*_*x*_,_FF_–*k*_*x*_,_FF_)=0, thereby eliminating horizontal phase mismatch by symmetry.

In the vertical (*z*-) direction, phase matching plays a negligible role due to the extremely small thickness of the nonlinear medium, on the order of tens of nanometers. Under these conditions, vertical phase mismatch is effectively tolerated and does not significantly limit the overall SHG efficiency.

As illustrated in Figure 1, the structure exhibits mirror symmetry with respect to the central *y-z* plane. This symmetry allows the counter-propagating pumps to selectively excite either even or odd photonic eigenmodes, depending on their relative phase. When the pumps are in phase, only even modes are excited, whereas a π-phase difference leads to the excitation of odd modes. This symmetry consideration becomes particularly important near the photonic BE, where field localization is maximized. Since the BE resonance corresponds to an even-parity mode, in-phase pumping is employed to ensure optimal coupling to this resonant state.

Figure 3 shows the spatial distribution of the electric field’s y-component, normalized to the incident field amplitude, i.e., |*E*_*y*_|/|*E*_*0*_|, for two representative cases. The top views (Figures 3a and 3c) represent the field in the *x-y* plane intersecting the MoS_2_ elements at midthickness, while the side views (Figures 3b and 3d) show the field in the *x-z* plane through the center of the MoS_2_ rods.

**Figure 3:**
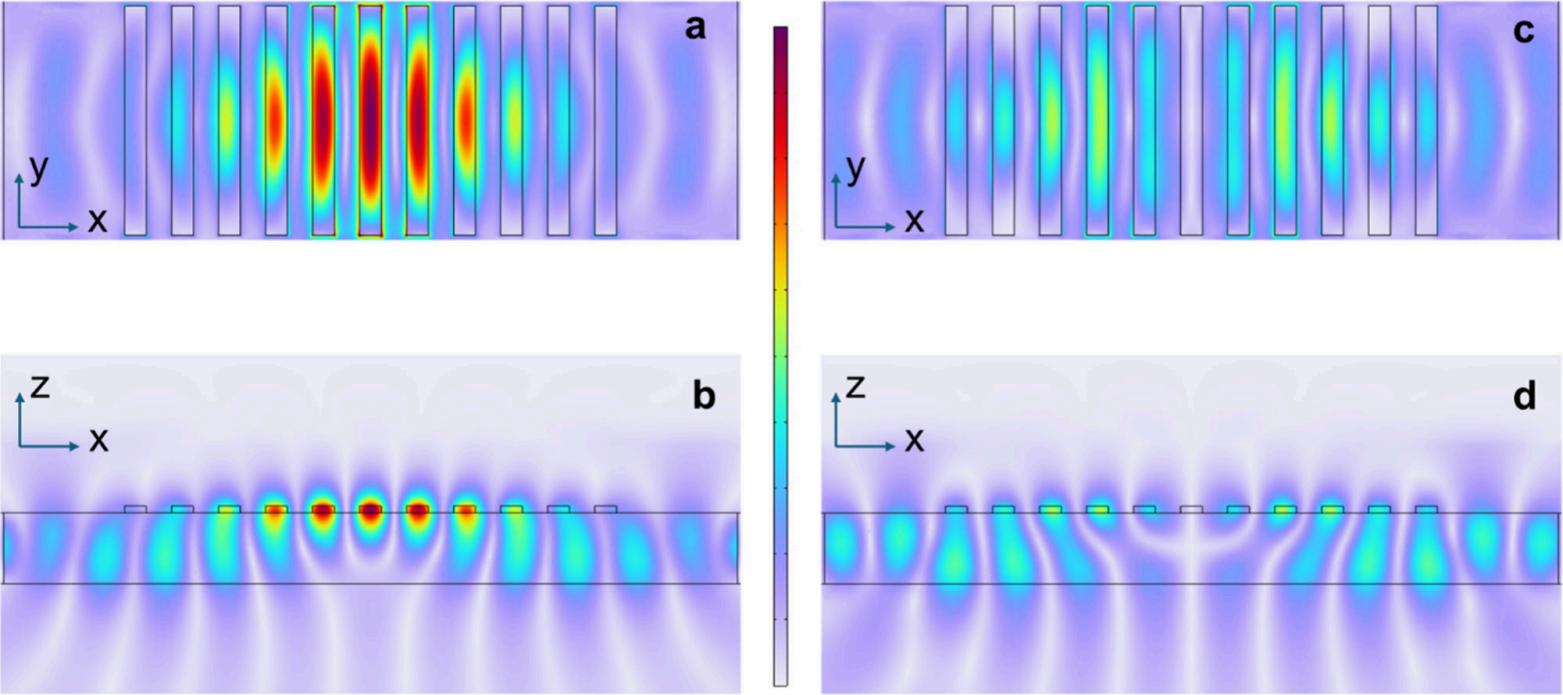
Spatial distribution of the normalized electric field amplitude |*E*_*y*_|/|*E*_*0*_| in a Si_3_N_4_ waveguide with 11 top-mounted MoS_2_ rods (95 nm wide, 30 nm thick) separated by 110 nm gaps. Panels (a, b): In-phase pump excitation (Δϕ=0) at 890 nm resonantly couples to the even-parity band-edge mode, yielding strong field confinement at the center of the structure. Panels (c, d): Out-of-phase excitation (Δϕ=π) leads to destructive interference at the mode center, suppressing coupling and resulting in weak, antisymmetric field profiles. (a, c) show horizontal cross sections in the *x–y* plane at mid-MoS_2_ height; (b, d) show vertical cross sections in the *x–z* plane through the rod axes. All maps share the same color scale.

In Figures 3a and 3b, the two pumps at 890 nm are in phase (Δϕ=0) and tuned to the BE resonance of an 11-period structure consisting of MoS_2_ stripes (95 nm wide, 30 nm thick) separated by 110 nm gaps. The resulting even-parity mode exhibits strong spatial confinement near the center of the structure, consistent with the expected profile.

In contrast, Figures 3c and 3d show the case of out-of-phase pumps (Δϕ=π) at the same wavelength and in the same structure. The odd-parity excitation is inefficiently coupled to the even-parity BE mode, resulting in significantly reduced field enhancement. The field distribution, characterized by a central node and two symmetric intensity maxima on either side, reflects the antisymmetric nature of the excitation and confirms the phase-selective mode coupling enabled by the pump configuration. The same color scale is used for all plots. For in-phase excitation, a peak field amplitude enhancement factor of approximately 5 is observed in the central MoS_2_ rod.

### Bloch Mode Analysis

Bloch mode analysis provides a powerful framework for designing light-matter interactions, even in finite periodic structures, due to several key advantages. First, the modes in finite structures often retain critical characteristics of their infinite periodic counterparts, including field confinement, resonance behavior, and modal symmetry. This makes the dispersion of Bloch modes an essential guide for the design of high-Q resonances, particularly those near the BE, where the group velocity approaches zero, enhancing local field intensities.^32^ This is especially relevant for nonlinear optical processes,^28,29,33,34^ where the spatial distribution and symmetry of the electromagnetic fields play a crucial role in determining the efficiency and selectivity of the interaction. In particular, the symmetry properties of Bloch modes allow for the selective enhancement or suppression of specific optical transitions, facilitating the design of resonant structures with tailored nonlinear responses.^35^ Moreover, Bloch analysis provides critical insights into mode coupling to free-space and guided modes, which is essential for optimizing nonlinear interactions that require both strong pump confinement and efficient signal extraction.^35–37^

For the structure under consideration, which is periodic along the *x*-axis, the Bloch analysis relies on a numerical eigensolver that discretizes the three-dimensional volume of the unit cell. This approach must account for both the chromatic dispersion and anisotropic response of all constituent materials, including the uniaxial MoS_2_, which exhibits strong extinction and dispersion in the visible and near-infrared ranges. To this end, we utilize the COMSOL Multiphysics electromagnetic eigensolver, imposing periodic boundary conditions along the *x*-axis as required by Bloch’s theorem:

$$E^{f}\left(r+\Lambda \mathrm{x̂}\right)=E^{f}\left(r\right)e^{iK_{B}\Lambda }$$

where **E** ^*f*^(***r***) is the mode’s field, periodic along *x̂* with periodicity Λ, and *f* indicates the discrete set of complex eigenfrequencies of the system. Since the structure can support modes that radiate into the surrounding air and substrate (typically glass), perfectly matched layers (PMLs) are implemented in the vertical direction to absorb outgoing radiation and prevent spurious reflections. We focus on periodic structures that show a BE for TE-like polarization (field mostly *y*-polarized) at λ_FF_ = 890 nm, corresponding, approximately, to a fundamental frequency *f*_FF_ = 337 THz. This choice of pump wavelength exploits the maximum peak of the χ_*yyy*_^(2)^ tensor element in MoS_2_, associated with the C exciton. In our design, we fix the Si_3_N_4_ waveguide dimensions and vary the periodicity Λ as well as the width *d*_*2*_ and thickness *t*_*M*_ of the MoS_2_ stripes.

The results are summarized in Figure 4a, which shows the combinations of (Λ *d*_*2*_) that position the BE of the periodic structure at the target frequency *f*_FF_ for three different values of *t*_*M*_. These combinations form curves in the (Λ *d*_*2*_) plane, with *d*_*2*_ generally decreasing as Λ increases. The precise shape of these curves is influenced by the significant chromatic dispersion of MoS_2_ near *f*_FF_, though a nearly linear relationship is observed for thinner stripes (e.g., *t*_*M*_ = 40 and 50 nm). These curves effectively map the parameter space in which a pump tuned to *f*_FF_ = 337 THz can excite the BE mode, resulting in significant local field enhancement. However, efficient second-harmonic generation (SHG) in this setup also depends on the radiation efficiency of the structure at the second-harmonic frequency *f*_SH_ = 2*f*_FF_ = 674 THz, which requires careful consideration of the vertical mode coupling.

**Figure 4:**
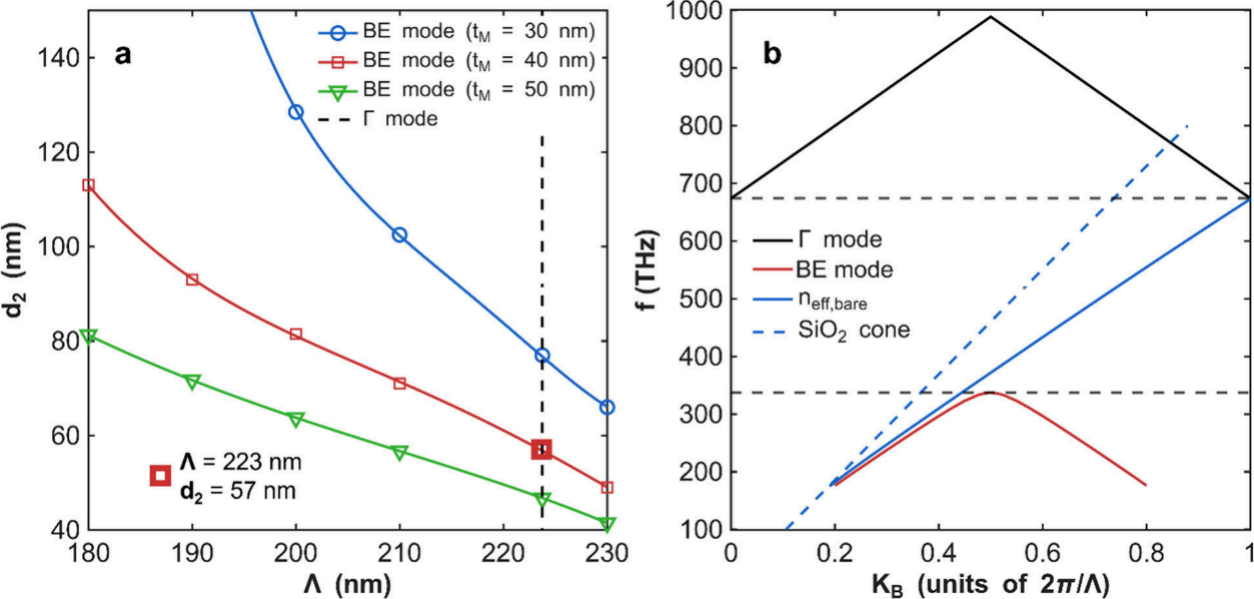
(a) Band-edge mode dispersion in the (Λ *d*_*2*_) space for three values of the MoS_2_ thickness (30, 40, and 50 nm). In all the points of the three curves, the eigenfrequency associated with the band edge mode is tuned at the target pump frequency *f*_FF_ = 337 THz. The vertical dashed line at Λ = 223 nm identifies the structures that excites the Γ mode at the SH frequency, *f*_SH_ *=2f*_FF_. (b) Dispersion of the BE and the Γ mode in the momentum-frequency plane. The band edge of the structure occurs at the target frequency *f*_FF_ – see the red curve corresponding to the BE mode. The target frequency is reported as a horizontal dashed line at 337 THz, while the Γ mode is tuned at the target SH frequency (indicated as a dashed horizontal line at 674 THz).

Efficient vertical emission is expected when SH light couples to a mode of the periodic structure at the Γ point (K_B_ = 0). This mode should be mostly polarized in the *y*-direction, in order to match the polarization selectivity imposed by the χ_*yyy*_^(2)^ tensor element which couples a *y*-polarized pump field to a *y*-polarized SH field, and it must possess even parity inside the unit cell, so that coupling of radiation in the vertical direction is allowed. It is important to notice that, owing to the high absorption losses in MoS_2_ at the SH frequency (the complex index of refraction of MoS_2_ at the SH frequency is ≈ 4.75+*i* 3.35), modes that show strong field confinement in the MoS_2_ stripes and that maximize the overlap integral of the SH mode with the FF mode in the nonlinear material are not necessarily optimal in the structure under investigation. In fact, while the maximization of the mode overlap integral leads to the maximization of SHG conversion efficiency in systems that are transparent for both the pump and the SH, it is reasonable to expect large SH absorption, at the expenses of SH radiation, in the structure under investigation. Therefore, a good condition for efficient SH emission, is the lattice-matching to the bare Si_3_N_4_ waveguide mode at the SH frequency. This condition is realized when $\beta_{1}=\frac{2\pi }{\lambda_{SH}}n_{1}\left(\lambda_{SH}\right)\approx \frac{2\pi }{\Lambda }$ , which translates into a requirement for the periodicity $\Lambda \approx \frac{\lambda_{SH}}{n_{1}\left(\lambda_{SH}\right)}$ . In the Bloch analysis of the unit cell of the 3D structure, this condition occurs as a *y*-polarized mode that crosses the Γ point with an eigenfrequency equal to *f*_*SH*_ and with a modest field localization inside the MoS_2_ stripes. The dispersion of this mode, labeled as Γ mode, appears as a vertical dashed line at Λ ≈ 223 nm in Figure 4a.

Simultaneous excitation of the BE mode at the FF and the Γ mode at the SH frequency occurs at the crossing points shown in Figure 4a. We expect maximum peaks of SHG conversion efficiency near these crossing points, in which the doubly resonance condition is realized. For one of the points, namely the one in which *t*_*M*_ = 40 *nm*,*d*_2_ = 57 *nm*,Λ = 223 nm, we have calculated the dispersion diagrams in the momentum-frequency space. The results are illustrated in Figure 4b. The BE mode shows its typical band edge curvature in the X point of the photonic crystal, i.e., $K_{B}=\frac{\pi }{\lambda }$ with a frequency tuned at the target value *f*_FF_ = 337 THz. At the same time the Γ mode crosses the Γ point (*K*_*B*_ = 0) at the target SH frequency 2*f*_FF_. The spatial profiles of the BE mode and the Γ mode are shown in Figure 5. The highly localized nature of the BE mode and, at the same time, the leaky nature of the Γ mode in the vertical direction, allow to exploit the inherently large nonlinearity of MoS_2_ by taking advantage of the strong pump-field enhancement at the BE with minimal absorption losses at the SH.

**Figure 5:**
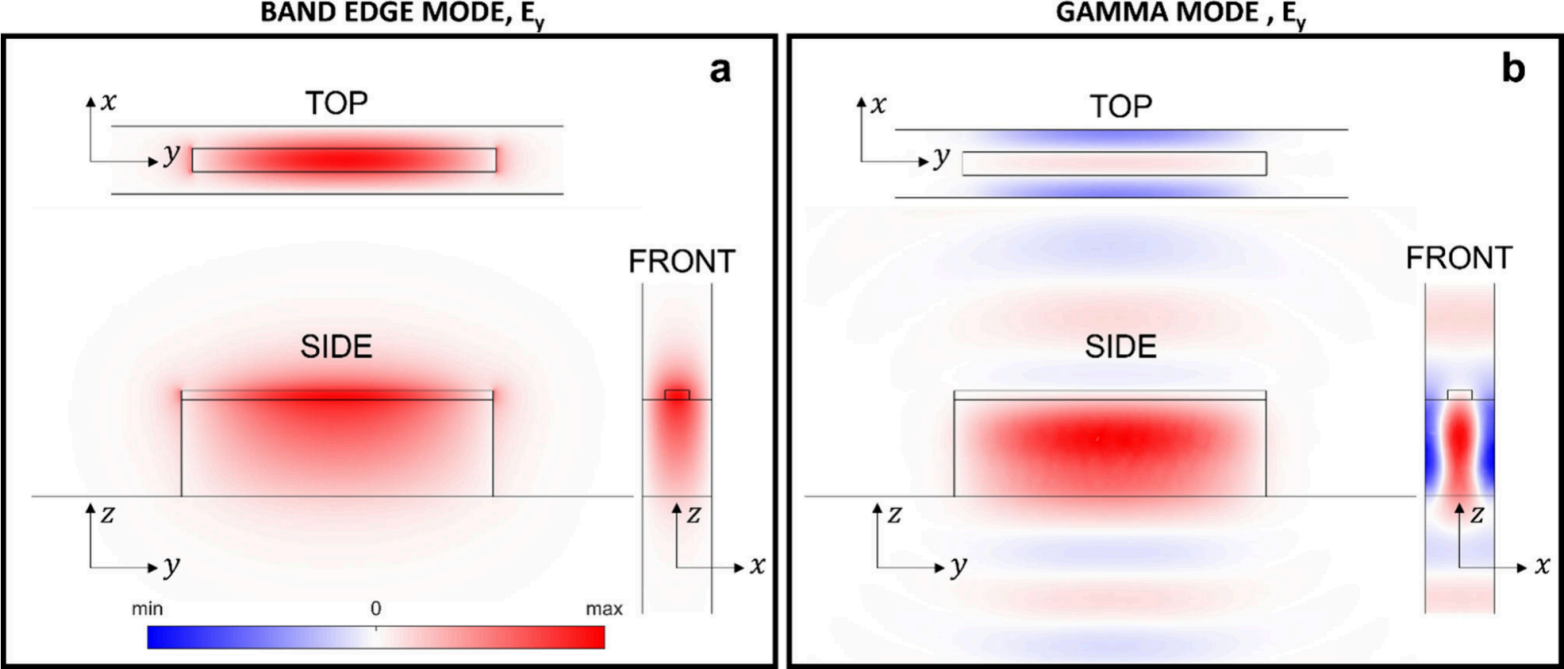
(a) Spatial distribution of the *E*_*y*_ field – here we report the real part of *E*_*y*_ in normalized units. The “top” cross section, in the *x-y* plane, is shown at the midplane of the MoS_2_ stripe in order to show the strong field confinement of the mode in the nonlinearly active portion of the unit cell. The “side” view, in the *y-z* plane, is taken in the midplane of the unit cell (x = 0), showing how the field extends also inside the Si_3_N_4_ region. The “front” view is taken in the *x-z* plane in the midplane of the unit cell. (b) Same as (a) for the Γ mode. The “top” view, taken in the middle of the MoS_2_ stripe, shows the modest localization in MoS_2_, while the side view illustrates the field localization in the Si_3_N_4_ region and the leakage in the vertical direction, both on the air side and substrate side. From the “front” view, the mode appears very similar to the TE-like mode supported by the bare waveguide, except for the vertical radiation leakage.

### SHG Numerical Simulations

Guided by both the analytical toy model and Bloch mode analysis, we identified a photonic resonance condition that enables efficient overlap between the fundamental BE mode at the pump frequency and the Γ-point mode at the second harmonic (SH). For a pump wavelength of 890 nm, this condition is satisfied for a unit cell period of approximately Λ ≈ 220 nm. As shown in Figure 2, strong field localization at the BE typically occurs for structures comprising 10 to 20 periods. An exception is found for the thinnest MoS_2_ layers (e.g., 20 nm), where the reduced index contrast requires longer structures (∼30 periods) to reach resonance. However, further increasing the number of periods in this regime provides only marginal improvements in effective interaction volume, while offering limited additional field confinement, thus leading to diminishing returns. Moreover, excessively long structures elongate the SH emission region along the propagation axis, resulting in asymmetric (elliptical) far-field spot shapes. Although increasing the waveguide width could help restore circular symmetry in the far field, this comes at the cost of a larger mode area and reduced field intensity, ultimately weakening the nonlinear interaction. Therefore, we focus our analysis on MoS_2_ thicknesses of *t*_*M*_ = 30, 40, and 50 nm, and adopt an 11-period structure as a practical trade-off between nonlinear enhancement and device compactness.

The χ^(2)^ value used in our calculations (800 pm/V) is taken from literature,^14^ where both experimental measurements and DFT-based predictions are in close agreement. In that study, the MoS_2_ flakes were obtained by mechanical exfoliation from high-quality 3R-phase single crystals commercially available from HQ Graphene (www.hqgraphene.com). For nanopatterned MoS_2_, aggressive dry etching processes, such as high-power reactive ion etching, are generally expected to introduce lattice defects and affect the value of the nonlinear susceptibility. However, the most recent experimental study employed a gentle two-step fabrication procedure combining top-down lithography with anisotropic wet etching.^16^ This method allows precise nanopatterning of 3R-MoS_2_ while preserving its high crystallinity and nonlinear optical response. Starting from a high-quality flake and using this low-damage etching process, the χ^(2)^ value remains effectively unchanged. The reported SHG measurements show excellent agreement with theoretical predictions, confirming the validity of 800 pm/V in the C-exciton band. These results fully support our use of the same χ^(2)^ value for the exfoliated 3R-MoS_2_ flake after nanopatterning.

We performed full-wave simulations using the frequency-domain module of COMSOL Multiphysics at a fixed pump wavelength of 890 nm. The calculations were carried out under the undepleted pump approximation, which is highly accurate in our regime, where the total conversion efficiency remains below 1%. In this regime, SHG scales with |χ^(2)^|^2^; our results can therefore be easily rescaled if a different value of χ^(2)^ is assumed, for instance in the case of MoS_2_ nanostructures with lower crystallinity due to defects or more aggressive fabrication processes. We first solved the linear field pattern for the FF (ω) wavelength and used it to compute the second-order nonlinear polarization acting as the source term for the SH field:

5 $${\mathrm{P⃗}}_{2\omega }^{NL}\left(x,y,z\right)=\varepsilon_{0}{\mathrm{\chi ̂}}^{\left(2\right)}\left(x,y,z\right){\mathrm{E⃗}}_{\omega }\left(x,y,z\right)\text{:}{\mathrm{E⃗}}_{\omega }\left(x,y,z\right)$$

where ε_0_ is the vacuum permittivity, *χ̂* ^(2)^ is the second order nonlinear susceptibility tensor, and *χ⃗*_*ω*_ is the field distribution at the FF. In our system, *χ̂* ^(2)^ is nonzero only within the MoS_2_ rods. The nonvanishing components of the tensor are dictated by the crystal symmetry.^14^ For a y- polarized pump field, the dominant nonlinear contribution arises from the χ_*yyy*_^(2)^ tensor element, which has been reported to reach values as high as 800 pm/V. The computed nonlinear polarization distribution *P⃗*_2ω_^*NL*^ (*x*,*y*,*z*) was then used as the input source in COMSOL Multiphysics, which solves the full inhomogeneous wave equation to obtain the SH field distribution throughout the simulation domain.

The SH output power was finally calculated by integrating the time-averaged Poynting vector over a collection plane located 400 nm above the Si_3_N_4_ waveguide. Emission toward the SiO_2_ substrate was also evaluated. Due to the vertical symmetry of the structure, SHG emission is found to be nearly symmetric in both upward and downward directions. To achieve unidirectional emission, additional design strategies, such as incorporating distributed Bragg reflectors (e.g., TiO_2_/SiO_2_ stacks) or metallic back-reflectors (e.g., Au) in the SiO2 substrate could be employed to suppress the downward emission and redirect SHG upward.

## Results and Discussion

### SHG Efficiency Analysis

A systematic parametric sweep was performed over the design space defined by the MoS_2_ stripe width (*d*_*2*_) and the unit cell period (Λ) from the previous analysis. For each MoS_2_ thickness (*t*_*M*_ = 30, 40, and 50 nm), we carried out dedicated simulations for the 11-period configuration to assess the SHG efficiency. The corresponding SH output powers are reported in Figure 6 as a function of (*d*_*2*_, Λ) under counter-propagating pump excitation, with each input beam delivering 1 W at 890 nm.

**Figure 6:**
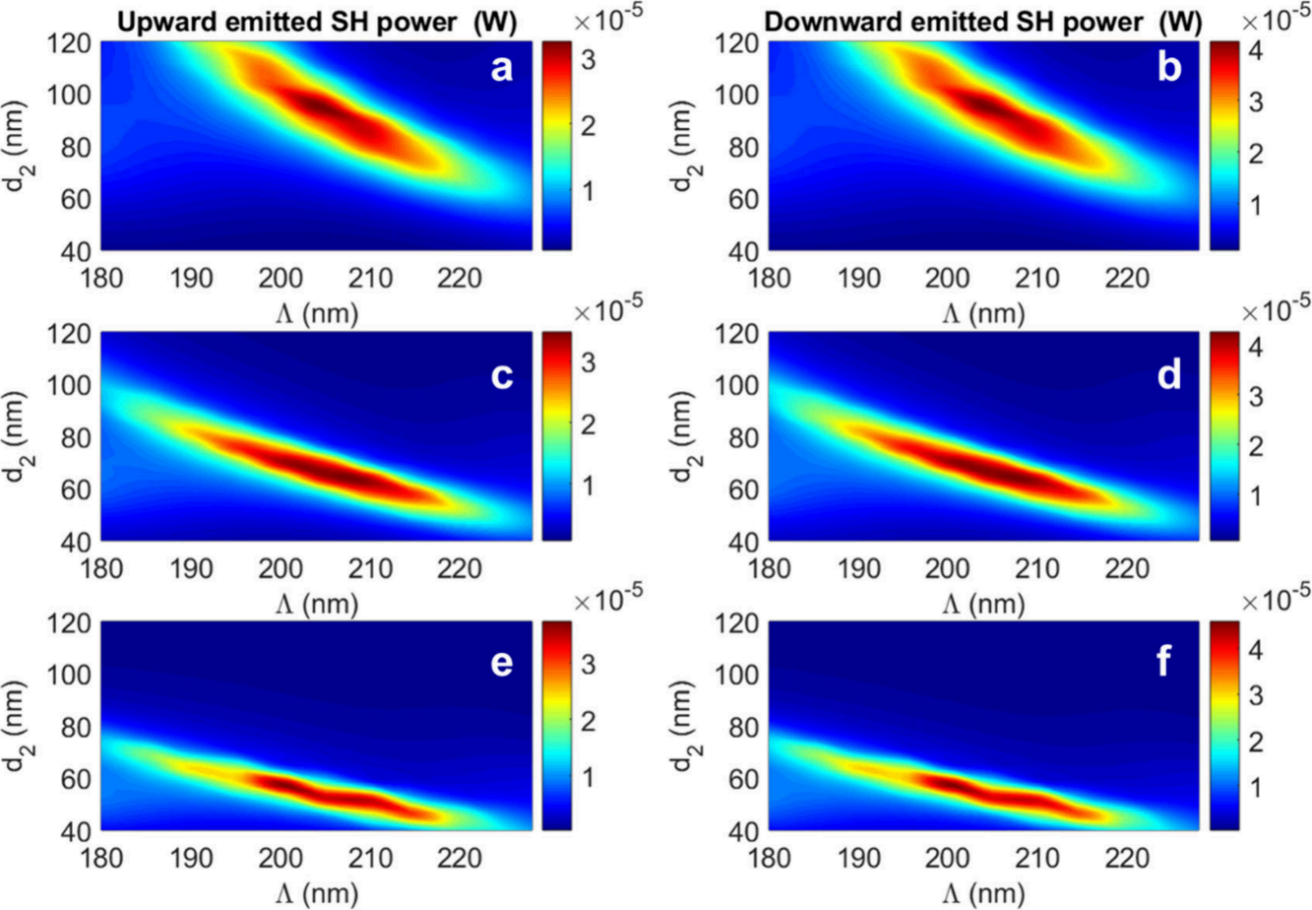
Emitted second-harmonic power *P*_*SH*_^+^ (*d*_2_,Λ)­under in-phase counter-propagating pump excitation for 11-period MoS_2_ arrays; (a, c, e): upward-emitted power *P*_*SH*_^+^ for *t*_*M*_ = 30, 40, and 50 nm, respectively; (b, d, f) downward-emitted power *P*_*SH*_^‑^ for the same thicknesses.

It is worth noting that the Bloch mode analysis (see Figure 4) predicted optimal phase-matching conditions for Λ ≈ 223 nm and MoS_2_ stripe widths of approximately 87, 57, and 47 nm for *t*_*M*_ = 30, 40, and 50 nm, respectively. The full-wave simulations, performed on finite 11-period structures, yield slightly different optimal parameters, i.e. Λ ≈ 205 nm and stripe widths of 95, 64, and 57 nm, respectively. These small discrepancies are attributed to the finite size of the simulated structures: while the Bloch model assumes an ideal infinite periodicity, the finite structures exhibit a BE resonance that shifts with the number of periods.

We focus on the 30 nm thick MoS_2_ rods because they exhibit greater tolerance to fabrication variations (see the larger area in Figures 6a and 6b compared to Figures 6c–6f). Although slightly thicker layers (40–50 nm) provide marginal gains in efficiency due to improved field confinement and modal overlap, they lead to narrower MoS_2_ features per unit cell. This imposes stricter requirements on lithographic resolution and edge roughness control, making the fabrication process more challenging. We will discuss fabrication constraints and the robustness of our design in more detail in the next section.

For the 30 nm thick configuration, the maximum upward-emitted SH power reaches approximately 32 μW, with a comparable ∼ 42 μW directed downward, resulting in a total SHG efficiency of ∼ 0.004%. Given the waveguide’s mode area, this corresponds to a pump intensity of approximately 300 MW/cm^2^.

Figure 7 presents the numerically computed second-harmonic (SH) field distribution for the optimized device configuration, comprising MoS_2_ rods with a width of 95 nm, a thickness of 30 nm, and a 110 nm gap between adjacent elements. Panel (a) shows the SH field amplitude in the *x–y* plane at a height of 400 nm above the waveguide surface, illustrating a directional upward emission profile with limited lateral spread. Panel (b) displays the SH field amplitude in the *x–z* plane, revealing pronounced vertical emission in both upward and downward directions. The strong SH field observed inside the MoS_2_ rods originates from the intense pump field localization, which induces a correspondingly strong nonlinear polarization at the SH frequency. This polarization acts as a localized source term, launching SH radiation that couples efficiently to out-of-plane radiative modes, leading to pronounced vertical emission. A more detailed analysis of the SH field distribution at increasing distances from the MoS_2_ rods, showing the transition from near- to far-field, is provided in Figure S3 of the Supporting Information.

**Figure 7:**
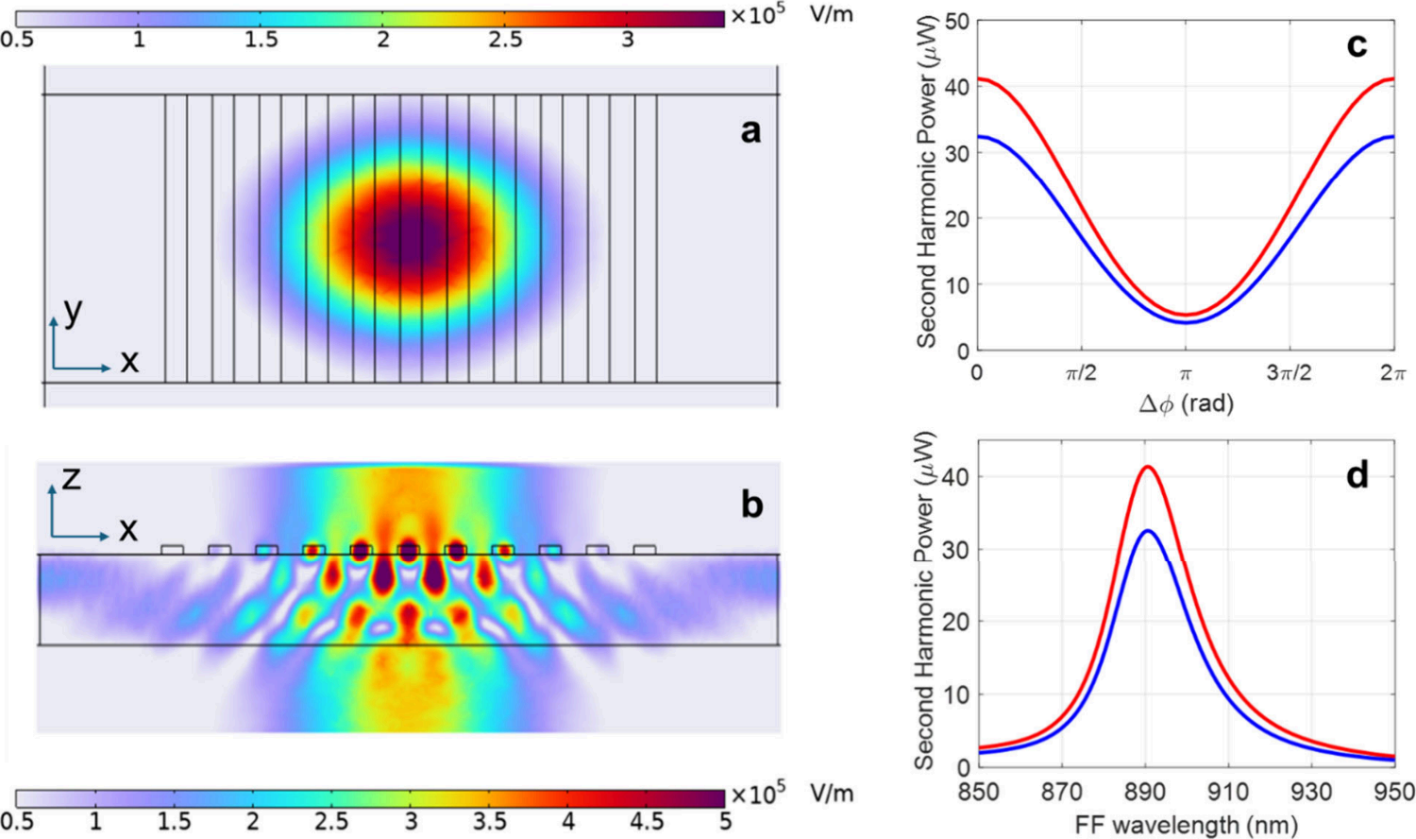
Full-wave numerical simulation of the second-harmonic response in the optimized MoS_2_ rod array (rod width = 95 nm, thickness = 30 nm, inter-rod gap = 110 nm). (a) Spatial map of the SH electric-field modulus in the plane parallel to the chip surface, at a height of 400 nm above the waveguide. (b) Cross-sectional distribution of the SH electric-field modulus through the waveguide midline (*x-z* plane), revealing almost balanced upward and downward radiation lobes and confirming efficient coupling into out-of-plane modes. (c) Phase-dependent upward- (blue line) and downward- (red line) radiated SH power plotted as a function of the relative phase delay Δϕ between two counter-propagating pump beams. (d) Spectral response of the upward- (blue line) and downward- (red line) generated SH power around the MoS_2_ C-exciton. The 20 nm fwhm bandwidth fully covers the spectral width of a 100 fs Fourier-limited pulse, ensuring broadband compatibility with ultrafast excitation.

Figure 7c demonstrates coherent control of the SHG process. For the selected structure, we calculate the upward- (downward-) emitted SH power as a function of the phase delay between the two counter-propagating pump beams. The resulting interference enables modulation of the generated SH signal with a contrast ratio of 10 between maximum and minimum SH output. This phase-sensitive response confirms the coherent nature of the SHG process in the distributed mode structure and can be exploited for dynamic switching or encoding applications.

We finally compute the SHG efficiency as a function of pump wavelength near the C-exciton resonance of MoS_2_, centered around 890 nm. Panel 7d shows that the device exhibits a broad spectral acceptance, with an enhancement bandwidth that fully encompasses the spectrum of a 100 fs Fourier-limited pulse (corresponding to ∼ 10 nm fwhm in wavelength). This confirms compatibility with ultrafast excitation.

### Feasibility and Robustness Analysis

As previously discussed, the 30 nm configuration offers an excellent compromise between nonlinear performance and fabrication robustness, as confirmed by the broader high-efficiency regions observed in the (*d*_*2*_ *, Λ*) parameter maps of Figure 6. These wider tolerance windows highlight the suitability of this design for scalable and reproducible nanofabrication.

The adoption of moderate-Q resonances is a deliberate design choice. Since MoS_2_ exhibits non-negligible absorption even at the fundamental wavelength, strong field enhancement would inevitably increase parasitic losses. For this reason, we favor broadband energy (BE) field buildup over narrow cavity-based resonances. The distributed mode structure enables coherent superposition of the SH fields generated within each MoS_2_ segment, without the need to confine the field inside a single high-Q resonator. This strategy provides a balanced trade-off among field enhancement, absorption control, and robustness to fabrication tolerances.

To assess fabrication robustness, we performed 50 full-wave simulations including Gaussian-distributed random variations (σ = 2 nm) simultaneously applied to all key geometrical parameters of each individual rod, including width, height, and position, thus inherently accounting for deviations from perfect periodicity across the entire array. In the same simulations, realistic surface and edge roughness were included, modeled with an RMS deviation of 2 nm and a correlation length of 26 nm, thus capturing scattering-induced perturbations directly affecting mode confinement.

Under these deviations, the SHG power remains highly stable, yielding average values of 25.7 μW (upward) and 33.0 μW (downward), with standard deviations of 3.5 μW and 4.4 μW, respectively. Compared to the ideal design (32 μW and 42 μW), these results indicate that fabrication tolerances and roughness-induced disorder can reduce the conversion efficiency by approximately 20%.

To isolate the effect of geometrical tolerances from that of surface and edge roughness, we performed an additional set of 50 simulations including only random variations of the geometrical parameters while neglecting roughness. The resulting average SHG powers, 27.5 μW (upward) and 35.1 μW (downward), with standard deviations of 2.8 μW and 3.6 μW, are statistically consistent with the previous analysis, corresponding to a 15% reduction in efficiency relative to the ideal case. Therefore, the inclusion of surface and edge roughness introduces a further, yet moderate, degradation of the SHG efficiency compared to that induced by geometrical fabrication tolerances alone.

It is worth noting that the adopted statistical parameters are representative of realistic fabrication conditions. The Gaussian fluctuations (RMS= 2 nm) accurately capture typical deviations in feature size, thickness, and periodicity achievable with current nanofabrication technologies, whereas the assumed surface and edge roughness (RMS= 2 nm) slightly overestimates the values reported for state-of-the-art MoS_2_ nanostructures. Indeed, ref 16 demonstrates the successful patterning of 20–25 nm-thick etched 3R-MoS_2_ nanostructures with excellent edge fidelity and surface quality, achieving subnanometer edge roughness described as “atomically precise.” Moreover, discrepancies remain negligible as long as the perturbation amplitude is well below the characteristic feature size.^38^ Therefore, we are confident that, under realistic fabrication conditions, the dominant source of performance degradation originates from dimensional tolerances (feature length, thickness, and periodicity deviations), whereas the impact of surface and edge roughness is minimal.

For completeness, we extended our study to a more demanding scenario by reducing the correlation length of the roughness profile to ∼ 18 nm (while increasing the RMS to 3 nm), thereby introducing a more granular morphology. This condition represents a regime in which surface and edge roughness begin to play a significant role. Also in this case, 50 full-wave simulations were carried out to ensure statistical convergence. The average SHG powers were 25.2 μW (upward) and 32.0 μW (downward), with standard deviations of 3.9 μW and 4.9 μW, respectively. Representative meshing configurations illustrating typical device realizations under Gaussian perturbations are shown in Figure S4a–b. As shown in Figure S4b, this case intentionally overestimates the roughness and represents an upper-limit scenario, where disorder effects become more evident (conversion efficiency reduced by ∼ 25%).

In conclusion, our robustness analysis shows that, under realistic fabrication conditions, dimensional tolerances and roughness together are expected to maintain about 80–85% of the ideal conversion efficiency.

Regarding thermal and long-term stability, MoS_2_ exhibits excellent environmental robustness, with structural degradation only above ∼ 400 °C.^39^ In our configuration, the MoS_2_ layer is embedded within a Si_3_N_4_ waveguide, which efficiently dissipates heat thanks to its high thermal conductivity. The use of femtosecond pumping further mitigates thermal load due to the low duty cycle. Literature reports indicate that MoS_2_ nanostructures withstand peak intensities up to 100–300 GW/cm^2^ without damage,^40^ consistent with experimental conditions in reference.^15^ In our case, efficient SHG is already achieved at moderate intensities (∼300 MW/cm^2^), and conversion efficiencies up to 0.4% are reached at 30 GW/cm^2^, safely below known damage thresholds. These aspects confirm suitability for stable long-term operation in nonlinear photonics.

Finally, efficient fiber-to-chip coupling can be obtained using tapered ridge waveguides and grating couplers at both device facets, with typical efficiencies around 50%, potentially improvable via more advanced schemes.^41^

### Performance Benchmarking and Outlook for Future Developments

The simulated conversion efficiency of ∼ 0.004% at an input pump peak power of 1 W (corresponding to a waveguided mode intensity of approximately 300 MW/cm^2^) for the 30 nm configuration stems from the intrinsically large nonlinear susceptibility of 3R-MoS_2_ near the C-exciton resonance, combined with the extremely reduced nonlinear interaction volume and a hybrid waveguide design that enables strong field confinement while maintaining a relatively low Q-factor (∼10). Such configuration ensures efficient evanescent coupling of the pump field into the high-χ^(2)^ MoS_2_ layer while mitigating SH absorption at the C-exciton resonance, ultimately enabling an extremely compact device footprint on the order of 1 μm^2^.

Recent work reported four to five orders-of-magnitude enhancement in SHG from precisely etched 25 nm-thick 3R-MoS_2_ metasurfaces:^16^ in that study, the pump was tuned to a quasi-bound state in the continuum (Q-BIC), while the generated second harmonic lies in the C-exciton spectral range. While this currently represents the best performance achieved in nanopatterned 3R-MoS_2_ platforms, it relies on resonances with Q-factors ≳100, making the device sensitive to fabrication tolerances. Although the comparison is not direct, since the scheme proposed by Zograf et al.^16^ operates in a free-space configuration with a focused pump and collects the SH signal in reflection, whereas our device relies on guided-wave pumping in a ridge-waveguide platform, both approaches share the same overarching goal: maximizing the nonlinear response of an ultrathin 3R-MoS_2_ nanostructured layer while mitigating absorption losses. Moreover, their metasurface architecture represents, to date, the most efficient SHG performance demonstrated in nanopatterned 3R-MoS_2_ films. We therefore consider their results^16^ as the most suitable benchmark to objectively contextualize the performance of our platform, while fully acknowledging the fundamental differences between the two operating regimes.

On these grounds, we introduce the following Nonlinear Figure of Merit (NFM), defined as NFM = η/(P_ω_ · V_NL_), where η is the SHG conversion efficiency, P_ω_ the pump peak power, and V_NL_ the employed nonlinear material volume. This figure of merit is intended to objectively assess the nonlinear performance of ultrathin χ^(2)^ nanostructures in integrated-photonics applications, where the interaction volume is severely limited and each element must operate efficiently under reduced pump power conditions. In such scenarios, maximizing the nonlinear response per unit power and per unit material volume becomes essential, enabling dense on-chip integration without relying on high-Q resonances or long interaction lengths. Using the efficiency values extracted from the robustness analysis, our structure yields an NFM ranging from 5.9 × 10^8^ W^1–^ cm^–3^ in the ideal case to 4.4 × 10^8^ W^1–^ cm^–3^ in the worst case scenario evaluated including fluctuations in the geometrical parameters and overestimated surface and edge roughness.

The comparison with the performance reported in^16^ is based on their published data. We consider a reported conversion efficiency of 0.08%, obtained with a 0.3 mW average pump at an 80 MHz repetition rate and 100 fs pulse duration, corresponding to a peak power of 37.5 W. Using the reported peak pump intensity of 5 × 10^7^ W/cm^2^, the effective spot area is 7.5 × 10^–7^ cm^2^. With a 25 nm film thickness and a postetch volume-filling factor of approximately 0.6, their estimated nonlinear volume yields an NFM of about 1.9 × 10^7^ W^1–^ cm^–3^. Thus, our device exhibits a ∼ 25× higher NFM if realistic fabrication tolerances and surface and edge roughness are included in the design. We emphasize that this comparison is not definitive, as it contrasts theoretical predictions (even including fabrication-imperfection effects) with experimental results. Nevertheless, it underscores the remarkable efficiency with which our design harnesses the nonlinear material.

Furthermore, the strong SHG performance is indicative of the potential of the same architecture for SPDC,^42,43^ due to the reciprocal nature of the two χ^(2)^ processes. Recent studies have already reported SPDC in MoS_2_-based devices that were previously investigated and benchmarked via SHG.^15,19^ While a rigorous analysis lies beyond the scope of this work, our findings suggest that the proposed SHG-optimized architecture could also operate efficiently in the SPDC regime.

More specifically, a high-frequency focused pump (e.g., λ ≈ 445 nm) exciting the patterned MoS_2_ regions could generate down-converted photon pairs (λ ≈ 890 nm) directly into the fundamental TE-guided mode of the Si_3_N_4_ waveguide. This emission geometry enables seamless injection of quantum states into on-chip photonic circuitry without requiring external filtering, alignment, or collection optics. Multiple such elements could be integrated in a compact footprint, appropriately phased and synchronously pumped, to increase pair-production rates without increasing pump intensity and thus mitigating multiphoton events.

A complete quantitative evaluation of photon-pair brightness, spectral correlations, and quantum-state properties will be addressed in future work.

## Conclusions

In this work, we proposed a hybrid photonic architecture designed to enable efficient second-harmonic generation (SHG) in the C-exciton absorption band of 3R-phase MoS_2_. Despite the extremely strong absorption in this spectral region, our results show that it is possible to achieve significant SHG efficiencies by leveraging a meticulous interplay of multiple physical mechanisms: the field enhancement associated with photonic band-edge effects at the pump wavelength, a counter-propagating waveguided pumping scheme, the intrinsically large second-order nonlinear susceptibility of MoS_2_ at the C-exciton, and the resonant outcoupling of the second harmonic into a leaky optical mode.

This combination allows our structure to operate in a regime where standard phase matching or quasi-phase matching techniques are not viable, due to the extremely short attenuation lengths of visible SH signals in the C-exciton band, often limited to just a few tens of nanometers. According to our simulations, SHG conversion efficiencies approaching 0.1% can be achieved with peak pump intensities on the order of 10 GW/cm^2^ (i.e., a peak power of 33 W), all within a device footprint as small as 1 μm^2^, making it highly suitable for on-chip integration.

The demonstrated performance is particularly promising for applications in SPDC, where a tightly focused visible pump beam could generate entangled photon pairs directly into the guided modes of a silicon nitride (Si_3_N_4_) ridge waveguide. Operating around 890 nm, the photon pairs would lie within the optimal detection window of mature silicon single-photon detectors, enabling high detection efficiency with well-established, low-noise technology.

In conclusion, the proposed scheme demonstrates an effective approach to frequency conversion in the visible range via 3R-MoS_2_, while also providing a technologically compatible platform for the realization of compact sources of nonclassical light. This capability is particularly relevant for the integration of scalable quantum photonic circuits within CMOS-compatible architectures.

## Supplementary Material



## Notes

Squared modulus of the electric field normalized to the squared modulus of the incident field E0, for a representative case with *t*_*M*_ = 20 nm and different numbers of periods N; *W­(d*_*2*_ *,N)* as a function of the number of periods *N*, for selected values of *d*_*2*_ and *t*_*M*_; square modulus of the SH electric field evaluated in the *x*–*y* plane at different *z*-positions with respect to the top surface of the Si_3_N_4_ waveguide; representative meshing configurations illustrating a typical realization of the device when random Gaussian fluctuations are included (PDF)
