# Supplementary material for: Compact Second-Harmonic Generation in the C‑Exciton Band of 3R-MoS2 for Integrated Quantum Photonics
Source: ACS Photonics. 2025 Dec 15;13(1):1–13. doi: 10.1021/acsphotonics.5c01266 (PMC12787318; doi:10.1021/acsphotonics.5c01266)
Supplement: Supplementary file 1 [file ph5c01266_si_001.pdf]

Supporting Information

Compact Second-Harmonic Generation in the C-Exciton Band of $3R\text{-}MoS_2$ for Integrated Quantum Photonics

Alessandro Bile[1], Domenico de Ceglia[2], Daniele Ceneda[1], Maria Cristina Larciprete[1], Marco Centini[1,*].

1. Department of Basic and Applied Science for Engineering (SBAI), SAPIENZA, Università di Roma, Via Scarpa, 16, Roma 00161, Italy

2. Department of Information Engineering, University of Brescia, Via Branze 38, Brescia, 25123, Italy

*Email: marco.centini@uniroma1.it

5 pages, 1 cover sheet, Figure S1, Figure S2, Figure S3, Figure S4.

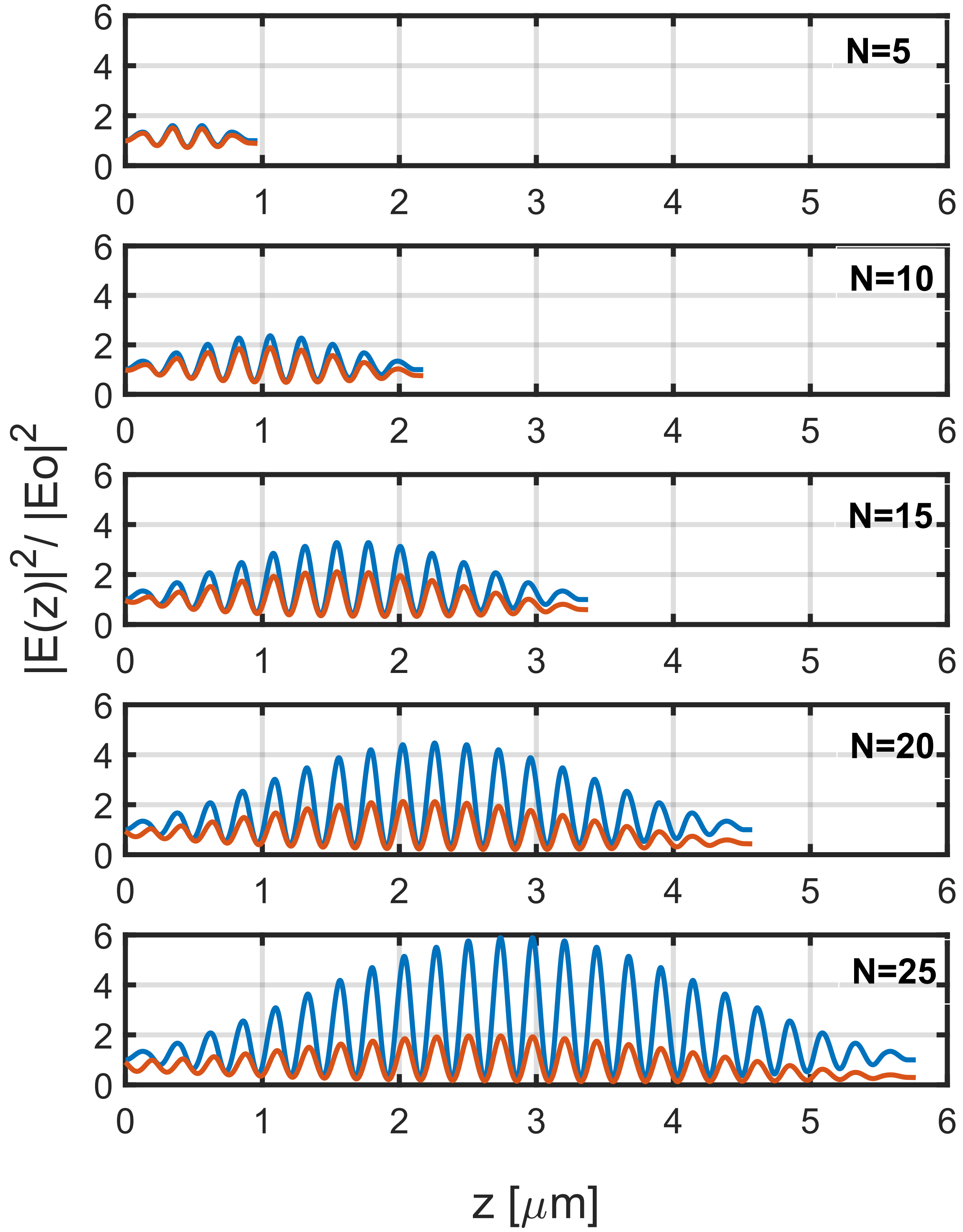

Figure S1: Squared modulus of the electric field normalized to the squared modulus of the incident field E0, for a representative case with t_M=20 nm and different numbers of periods N. The figure compares the case including optical absorption (red line) in the high-index material with the ideal lossless case (blue line).

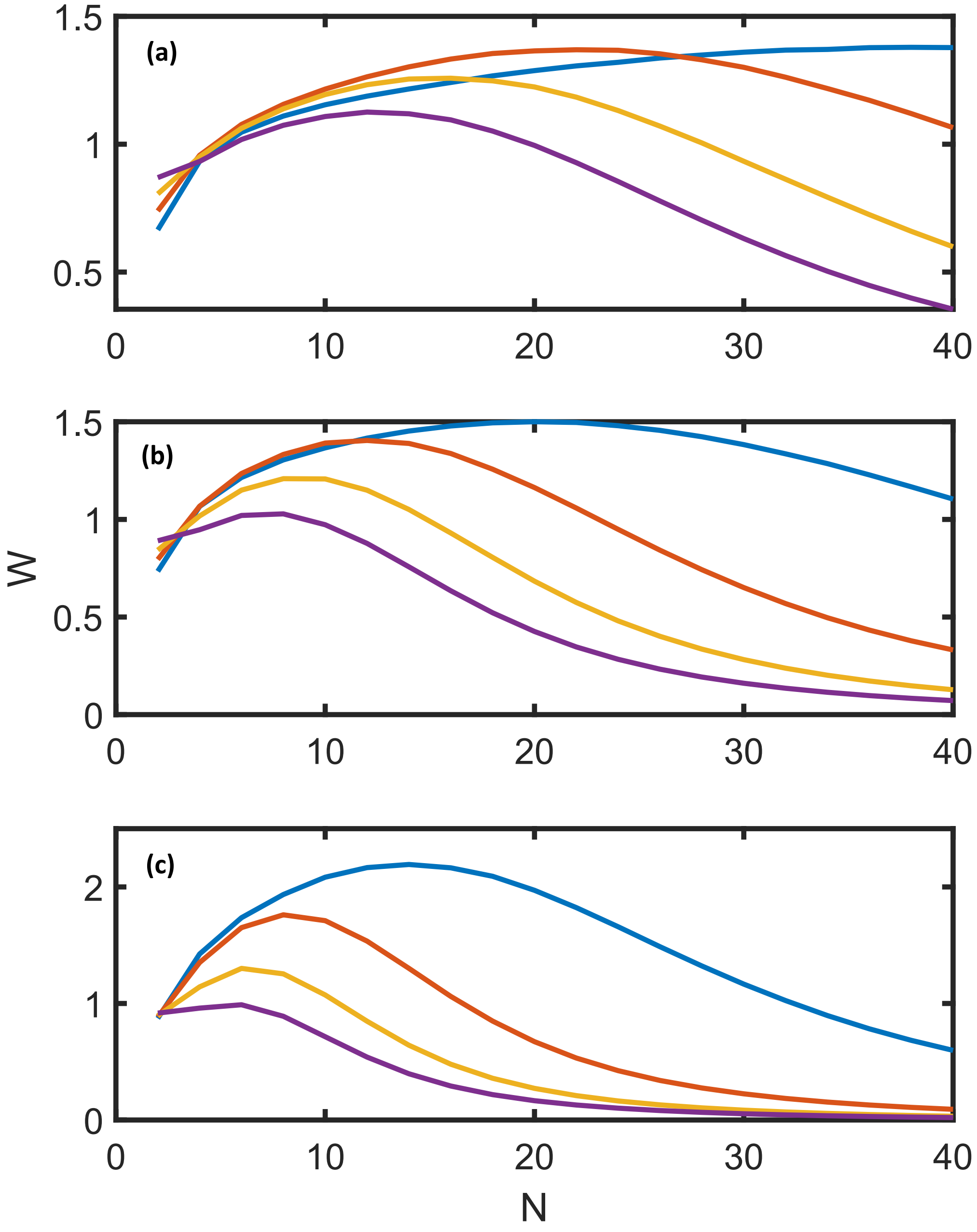

Figure S2: $W(d_2,N)$ as a function of the number of periods N, for selected values of d_2=20 nm (violet), 30 nm (yellow), 40 nm (red), 50 nm (blue) and three different MoS_2 thicknesses: (a) t_M=20 nm; (b) t_M=30 nm; (c) t_M=40 nm.

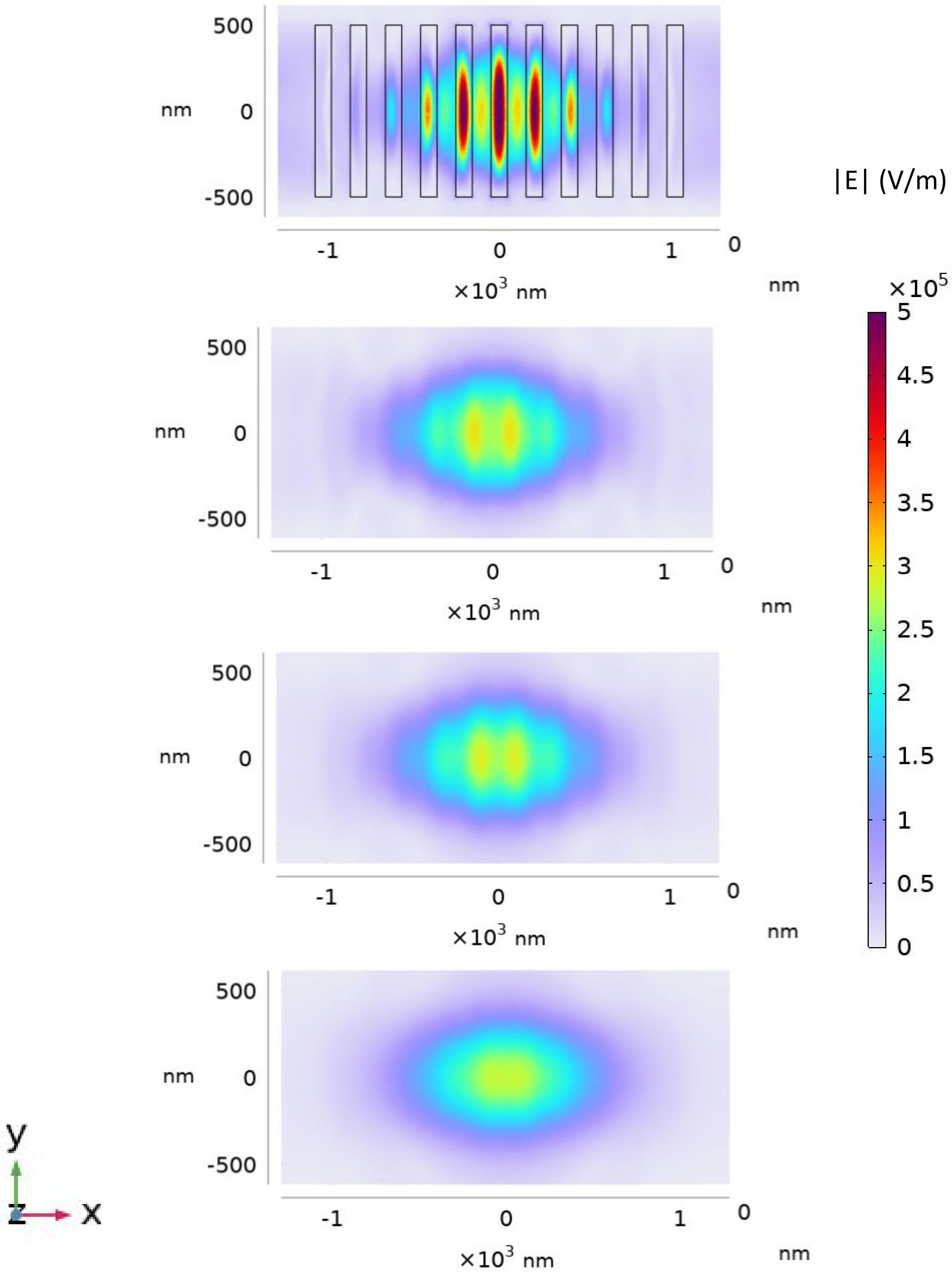

Figure S3: Square modulus of the SH electric field evaluated in the x–y plane at different z-positions with respect to the top surface of the Si_3N_4 waveguide: (a) z = 15 nm, corresponding to the middle of the MoS_2 rods; (b) z = 100 nm; (c) z = 200 nm; (d) z = 320 nm.

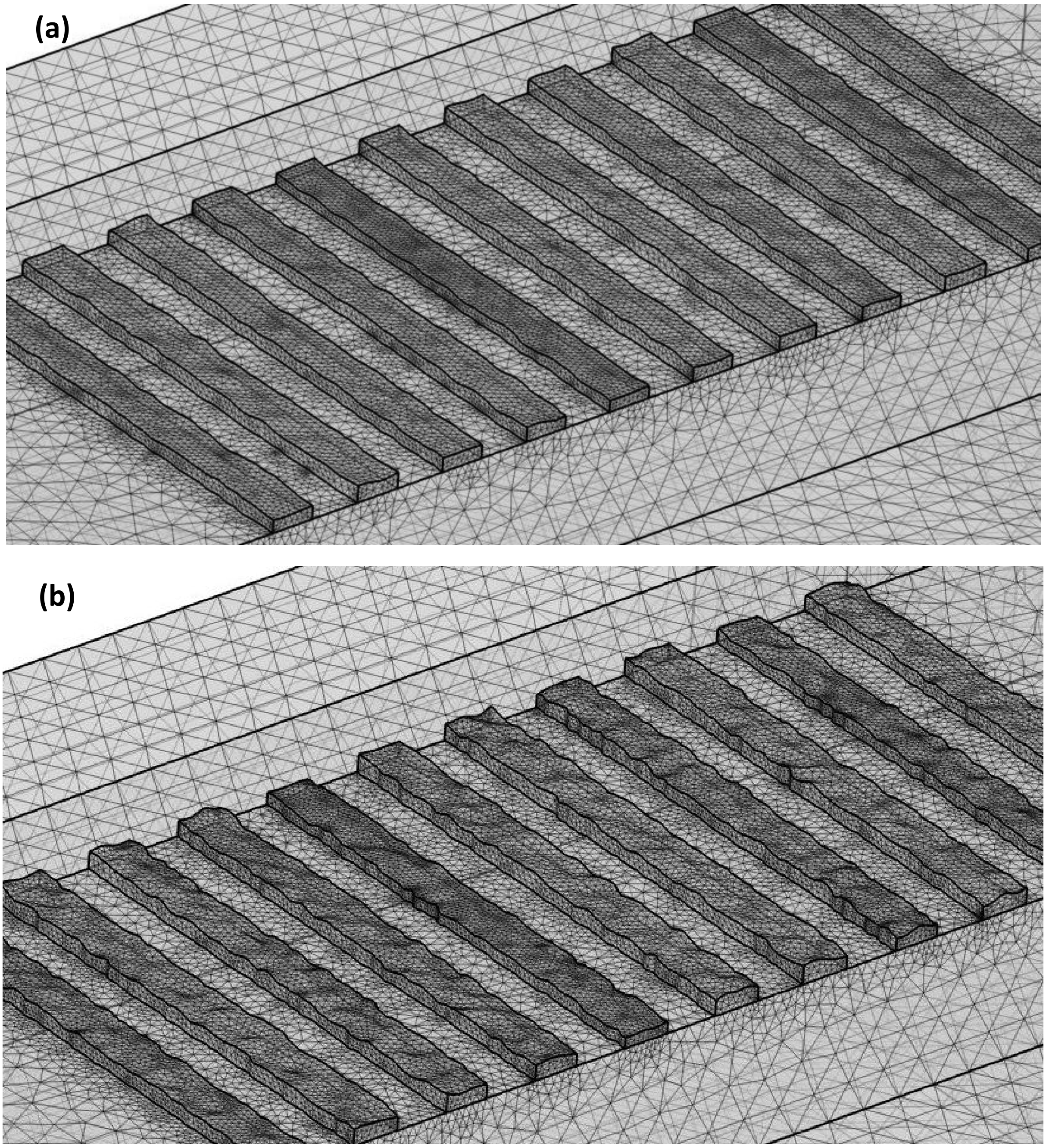

Figure S4: Representative meshing configuration illustrating a typical realization of the device when random Gaussian fluctuations are included. The rod average length, thickness, and spacing fluctuate around their optimal values following a Gaussian distribution with a standard deviation of 2 nm. In addition, surface and edge roughness are incorporated as Gaussian fluctuations around the average profiles, with: (a) standard deviation of 2 nm and a correlation length of 26 nm; (b) standard deviation of 3 nm and correlation length of 18 nm to obtain an overestimated granular morphology.
